# Expression and Immunological Characterization of African Swine Fever Virus *EP153R* Protein for Serodiagnosis and Its Delivery via a Recombinant PRRSV Live Vector

**DOI:** 10.3390/vaccines13111110

**Published:** 2025-10-29

**Authors:** Meng Luo, Wenna Shuai, Ziqiang Guo, Jiale Li, Liwei Li, Yanjun Zhou, Yifeng Jiang, Wu Tong, Yifan Zeng, Jinbin Wang, Li Zhao, Fei Gao

**Affiliations:** 1 Shanghai Veterinary Research Institute, Chinese Academy of Agricultural Sciences, Shanghai 200241, China; mengluo0223@outlook.com (M.L.); shuaiwenna2023@163.com (W.S.); guoziqiang0929@163.com (Z.G.); li.jia.le@foxmail.com (J.L.); liliwei@caas.cn (L.L.); zhouyanjun@caas.cn (Y.Z.); jiangyifeng@caas.cn (Y.J.); tongwu@caas.cn (W.T.); 15912708748@163.com (Y.Z.); aisi975@outlook.com (J.W.); 2 College of Veterinary Medicine, Yangzhou University, Yangzhou 225009, China; 3 College of Veterinary Medicine, Nanjing Agricultural University, Nanjing 210095, China; rna0426@126.com; 4 Ruipu Biological Co., Ltd., Tianjin 300308, China; 5 Jiangsu Co-Innovation Center for the Prevention and Control of Important Animal Infectious Disease and Zoonose, Yangzhou University, Yangzhou 225009, China

**Keywords:** African swine fever virus, pEP153R, recombinant protein expression, immunogenicity evaluation, polyclonal antibody production, serodiagnosis, indirect ELISA, PRRSV-based viral vector, reverse genetics, viral live vector

## Abstract

**Background/Objectives:** African Swine Fever (ASF), caused by the African Swine Fever Virus (ASFV), is a highly contagious and lethal disease in pigs, for which no recognized safe and effective vaccine is currently available. The ASFV *EP153R* gene, expressed during both early and late infection stages, exhibits strong protective potential. Utilizing advances in genetic engineering, recombinant PRRSV vector vaccines carrying ASFV exogenous genes were constructed. This study aims to prepare pEP153R-based polyclonal antibodies and an iELISA detection method using the constructed rPRRSV-EP153R as a specific target to verify the iELISA’s specificity and effectiveness. **Methods:** A prokaryotic plasmid, pCold-TF-EP153R, was constructed to express protein in BL21 (DE3). The purified soluble protein (2 mg/mL) was used to generate a murine polyclonal antibody and establish an indirect ELISA. The *EP153R* gene was inserted between ORF1b and ORF2a of PRRSV via reverse genetics, yielding recombinant rPRRSV-EP153R. Its biological properties were assessed in vitro and in vivo. **Results:** The pEP153R was specifically detected by both anti-His antibody and generated polyclonal antibodies. An established iELISA showed high specificity, sensitivity, and 98.18% accuracy. The antibodies specifically recognized pEP153R expressed in recombinant virus and eukaryotic systems. Additionally, the recombinant virus stably maintained *EP153R* without changes in virological characteristics relative to vHuN4-F112. In vaccinated piglets, the rPRRSV-EP153R induced a specific, consistent, and detectable immune response. **Conclusions:** The established iELISA, characterized by high specificity, sensitivity, and accuracy, furnishes reliable technical support for the serological diagnosis of ASFV. Meanwhile, the recombinant virus rPRRSV-EP153R demonstrates potential as a novel live vectored vaccine candidate, with the capability to induce specific immunity against both ASFV and PRRSV.

## 1. Introduction

African Swine Fever (ASF) is an acute, viral hemorrhagic and highly contagious porcine disease caused by the African Swine Fever Virus (ASFV). Highly virulent strains of ASFV can infect domestic pigs and wild boars, with a case fatality rate of up to 100% [1]. A total of 24 genotypes of AFSV have been identified. Among them, only genotypes I and II have spread from the African continent to other countries. The first ASF outbreak in China was detected in Shenyang in 2018, and the ASFV strain was identified as genotype II [2]. In 2021, the Harbin Veterinary Research Institute of the Chinese Academy of Agricultural Sciences first discovered a genotype I-attenuated strain [3]. In 2023, the research team isolated three recombinant strains of ASFV genotype I and genotype II [4]. After the introduction of ASFV into China, it rapidly spread across various regions, posing a serious threat to the domestic pig industry. Currently, the attenuated strains of ASFV have started to circulate. These strains have relatively low transmissibility, but the incubation period after infection is long, and the symptoms are not obvious, which increases the difficulty of ASFV detection and control. Naturally occurring inter-viral recombination has emerged, presenting a new challenge to the global pig industry. However, since there is no recognized safe and effective commercial vaccine for ASF, biosecurity measures remain a crucial strategy for the prevention and control of ASF.

ASFV is the sole double-stranded DNA virus with an icosahedral symmetric viral nucleocapsid, a capsule membrane, a genome size of approximately 170–190 kb, and the capacity to encode over 160 proteins [5]. Among these proteins, the one encoded by the ASFV *EP153R* gene is a non-structural protein that is expressed both in the early and late stages of viral infection [6]. Since pEP153R possesses a C-type lectin-like domain, it is referred to as the C-type lectin protein of ASFV. Given that macrophages, the target cells of ASFV, have receptors like the C-type lectin sequence, it is hypothesized that pEP153R is likely to be a potential antigenic protein with good protective properties [7]. Although the immune evasion function of pEP153R may also result in non-protective immune responses or antibody-dependent enhancement (ADE) effects, studies have demonstrated that it can inhibit cell apoptosis by influencing the activity of Caspase-3 and p53. Furthermore, this protein plays a role in viral immune evasion by down-regulating the expression of class I major histocompatibility complex (MHC-I) on the plasma membrane and modulating cell surface proteins. As such, it serves as a crucial immune evasion protein of ASFV [8]. Notably, numerous T-cell epitope mapping studies have indicated that pEP153R harbors a significant quantity of immunodominant epitopes that can be recognized by T cells from pigs that have survived ASFV infection [9]. This implies that introducing the corresponding gene into the host can effectively activate porcine T cells and imitate the cellular immune responses triggered by natural infection or attenuated live vaccination. Therefore, using this exogenous protein as a target antigen in vaccine design presents twofold advantages. Firstly, pEP153R itself acts as a T-cell target and actively participates in the immune response. Secondly, when expressed within the framework of a recombinant viral vaccine vector, the protein functions merely as an antigen and no longer possesses the immunosuppressive properties associated with ASFV. In essence, this strategy capitalizes on the antigenic potential of pEP153R while eliminating its pathogenic effects. Consequently, pEP153R shows promise as a candidate antigenic target for the development of vaccines against ASFV.

Porcine reproductive and respiratory syndrome (PRRS) remains one of the viral afflictions severely hampering the healthy progression of the global swine industry. It is caused by porcine reproductive and respiratory syndrome virus (PRRSV). Infected swine typically manifest reproductive dysfunctions, such as abortion and stillbirth in pregnant sows, along with symptoms like dyspnea and anorexia in piglets, and are prone to secondary infection [10]. PRRSV is an enveloped single-stranded, positive-sense RNA virus. Its genome length ranges from 14.9 kb to 15.5 kb. It belongs to the *Arteriviridae* family within the order *Nidovirales.* The PRRSV genome encompasses at least 11 open reading frames (ORFs) and is classified into two distinct genotypes: the European or type 1 genotype (also referred to as PRRSV-1) and the American or type 2 genotype (also known as PRRSV-2) [11]. In recent times, the PRRSV genome has undergone evolution, giving rise to novel, highly mutated, and recombinant strains. These emerging strains exhibit escalating virulence and are characterized by immunosuppressive properties and ADE [12]. Consequently, there is an urgent need to develop an effective PRRS vaccine capable of countering the ever-changing PRRSV. With the continuous progress of biotechnology in recent years, the development of live viral vectored vaccines by inserting exogenous genes into the genome of live viruses via genetic engineering techniques has shown considerable potential. Among diverse vector systems, the live attenuated PRRSV expressing exogenous ASFV antigens presents distinct combinatorial benefits. These advantages are mainly manifested in the following aspects: Firstly, PRRSV demonstrated high tropism for macrophages, which are also the primary target cells for ASFV infection and replication. Utilizing PRRSV as a delivery vehicle allows for the direct presentation of ASFV antigens to these crucial antigen-presenting cells, thereby significantly enhancing the efficiency and specificity of the immune response. Secondly, as a replicating vector, PRRSV promotes long-term and high-level expression of ASFV antigens in vivo. This results in continuous antigen exposure, which elicits a long-lasting immune response, notably superior to the transient immunogenicity provided by single-dose protein subunit vaccines. Thirdly, PRRSV vaccine technology, especially through the utilization of recombinant chimeric viruses, is relatively well-developed and has a well-defined safety profile. Therefore, this study aims to evaluate the expression efficiency of the ASFV pEP153R mediated by a PRRSV vector and to assess its potential as a candidate for developing a safer and more effective vaccine against African swine fever. Potential insertion sites for exogenous genes within the PRRSV vector include the non-essential regions of Nsp2, the coding region of ORF7, as well as the intergenic regions between ORF1b and ORF2a, ORF7 and 3′ UTR, and ORF4 and ORF5a. Among these, research has indicated that stable expression of foreign genes can be achieved by inserting them into a crucial site (between ORF1b and ORF2a) of the PRRSV genome, and the recombinant virus can be successfully rescued. For instance, the E2 gene of classical swine fever virus (CSFV) has been inserted into the intergenic region between ORF1b and ORF2 in a PRRSV vaccine strain to develop multivalent vaccines against PRRSV and CSFV [13]. Additionally, the granulocyte-macrophage colony-stimulating factor (GM-CSF) gene and the synthesized TRS6 sequence have been inserted between ORF1b and ORF2 of a PRRSV vaccine strain to generate a recombinant vaccine that can stimulate more robust immune responses and enhance the efficacy of vaccines against PRRSV infection [14]. However, research focuses on a PRRSV-vectored single-antigen (pEP153R) vaccine, which may have limitations in inducing full ASFV protection compared to multi-antigen platforms, though pEP153R’s T-cell epitopes and PRRSV’s macrophage tropism still support its value for future exploration.

Ultimately, to prepare polyclonal antibodies against the protein encoded by the ASFV *EP153R* gene and to investigate the heritability of the *EP153R* gene within the PRRSV live virus vector, this study employed the pCold-TF prokaryotic expression system for the prokaryotic expression of the *EP153R* gene. Polyclonal antibodies (pEP153R antibodies) were prepared by immunizing mice with purified pEP153R. The recombinant virus rPRRSV-EP153R was constructed and rescued using the live virus vector of the attenuated vaccine strain vHuN4-F112. The recombinant virus and the eukaryotic expression plasmid pCAGGS-EP153R constructed in our laboratory were verified using the prepared polyclonal antibodies, thus providing a reference for further exploration of the functions of the pEP153R. An immunization experiment demonstrated that rPRRSV-EP153R elicited a specific, consistent, and detectable immune response against PRRSV and ASFV antigens, highlighting its promise as a live vectored vaccine candidate.

## 2. Materials and Methods

### 2.1. Bioinformatics Analysis

The transmembrane (TM) regions of the ASFV pEP153R were analyzed and predicted using the online software TMHMM-2.0 (https://services.healthtech.dtu.dk/services/TMHMM-2.0/, accessed on 8 January 2025). The hydrophobicity or hydrophilicity profiles of pEP153R were analyzed using the online software ProtScale (https://web.expasy.org/protscale/, accessed on 8 January 2025). The B-cell epitopes within the pEP153R were predicted via the online tool (http://tools.iedb.org/main/bcell/, accessed on 8 January 2025).

### 2.2. Construction of Recombinant Vectors

**Figure 1:**
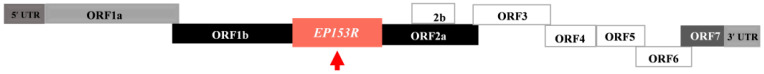
Schematic illustration of the construction of the recombinant plasmid pPRRSV-EP153R. Utilizing *Asc* I and *EcoR* V restriction sites, the *EP153R* gene fragment was inserted between ORF1b and ORF2a of vHuN4-F112 bone. The red arrow indicates the location of the *EP153R* gene.

The nucleotide sequence of the *EP153R* gene from the ASFV-SY18 strain was retrieved from the GenBank database (MH766894.3). An inactivated ASFV-positive nucleic acid sample served as the template for PCR amplification of *EP153R* gene using the primers listed in Table 1. The amplicons were generated using a high-fidelity DNA polymerase (New England Biolabs, Beijing, China, M0491) under optimized cycling conditions. The prokaryotic expression vector pCold-TF was double-digested with the restriction endonucleases *Xho* I and *Eco*R I (New England Biolabs, Beijing, China, R0146/R0101). PCR products and digested vector fragments were resolved via agarose gel electrophoresis (1% *w*/*v*) and purified using the QIAquick^®^ Gel Extraction Kit (QIAGEN, Hilden, Germany, 28704) according to the manufacturer’s instructions. The purified *EP153R* gene fragment was ligated into the linearized pCold-TF vector using ClonExpress II One Step Cloning Kit (Vazyme, Nanjing, China, C112). Recombinant plasmids were screened by restriction enzyme digestion, and positive clones were verified by sequencing (Beijing Qingke Biotechnology Co., Beijing, China). Sequences with 100% identity to the reference were designated pCold-TF-EP153R. Additionally, the intergenic region between ORF1b and ORF2a was selected as the insertion site for the ASFV *EP153R* gene, based on previous studies demonstrating its ability to support the stable expression of foreign genes without compromising viral viability [13,14,15]. The *EP153R* gene fragment was therefore inserted between ORF1b and ORF2a of the attenuated PRRSV strain vHuN4-F112 through *Asc* I and *EcoR* V (New England Biolabs, Beijing, China, R0558/R0195) restriction sites, as described in previous studies. Recombinant clones displaying the expected patterns were sequenced and confirmed as pPRRSV-EP153R. A schematic representation of the cloning is provided in Figure 1.

### 2.3. Prokaryotic Expression and Verification of Recombinant EP153R Gene Encoded with Protein (pEP153R)

The recombinant plasmid pCold-TF-EP153R was transformed into BL21 competent cells (TransGen, Beijing, China) using the heat-shock method. A single colony was inoculated into LB supplemented with ampicillin (100 μg/mL) and cultured overnight at 37 °C with shaking at 180 rpm. The overnight culture was diluted 1:100 in fresh LB containing ampicillin and grown until the optical density at 600 nm (OD600) reached 0.6–0.8. Protein expression was induced by adding IPTG (final concentration 1 mM), followed by incubation at 16 °C with agitation at 180 rpm for 20 h. Cells were harvested by centrifugation at 10,000× *g* for 10 min at 4 °C, resuspended in pre-chilled PBS (pH 7.4), and lysed by ultrasonication on ice (pulse on/off, 3 s/6 s, for a total sonication time of 20 min). The lysates were centrifuged at 10,000× *g* for 20 min at 4 °C to separate the soluble and insoluble fractions. Expression of the recombinant pEP153R was analyzed by SDS-PAGE and Western blotting using anti-6X His-tag^®^ antibody (Abcam, Cambridge, UK).

For protein purification, the soluble fraction was applied to a 2 mL His-tag Ni^2+^-NTA Agarose (QIAGEN, Hilden, Germany) pre-equilibrated with binding buffer. The column was washed sequentially with 5 mL of wash buffer containing increasing concentrations of imidazole (20 mM, 40 mM, and 60 mM). The bound recombinant protein was eluted with 5 mL of elution buffer containing 250 mM imidazole. Eluates were collected, pooled, and filtered through a 0.22 μm membrane (Millipore, Darmstadt, Germany) before downstream analysis. Meanwhile, the purity of the purified recombinant protein was assessed by densitometric analysis using ImageJ win64 software.

### 2.4. Preparation and Titer Determination of Polyclonal Antibody

Five four-week-old female BALB/c mice were used as experimental animals. Each mouse was immunized with 100 μg of the pEP153R. The immunization schedule consisted of three doses, and blood was collected from the orbital venous plexus two days after the final immunization. Serum was then isolated. Throughout the immunization process, the animals remained in good health. The pEP153R was diluted to a concentration of 2 μg/mL and stored at 4 °C overnight. Mouse serum antibodies were diluted with PBS at the following dilution ratios: 1:1000, 1:2000, 1:4000, 1:8000, 1:16,000, 1:32,000, 1:64,000 and 1:128,000. The titer of pEP153R polyclonal antibody was determined using ELISA.

### 2.5. Optimization of iELISA Conditions

The checkerboard titration method was employed to optimize the indirect ELISA (iELISA) conditions. The antigen was diluted in eight concentration gradients: 10, 8, 4, 2, 1, and 0.5 µg/mL. ASFV-positive and -negative sera were diluted according to six gradients: 1:40, 1:80, 1:100, 1:200 and 1:400. Antigen dilutions were prepared using carbonate buffer (CBS), phosphate-buffered saline (PBS), and Tris-HCl (pH 6.8). The antigens were coated onto ELISA plates for 1, 2, and 3 h at 37 °C and stored overnight at 4 °C. Additionally, blocking solutions containing 5% skim milk, 5% BSA, 2% skim milk, and 2% BSA in PBST (PBS with Tween 20) were used to block the plates. The blocking incubation was performed at 37 °C for 1, 2, and 3 h, respectively. The HRP-conjugated goat anti-pig IgG (H + L) secondary antibody (Abcam, Cambridge, UK) was diluted in PBST at concentrations of 1:2000, 1:4000, 1:8000, 1:10,000 and added to the plates. Through these steps, the optimal antigen coating concentration, serum dilution, coating condition, blocking conditions, and secondary antibody dilution were determined. Furthermore, the optimal antibody reaction times (30 min, 45 min, 60 min, and 75 min) and TMB substrate reaction conditions (37 °C for 5 min, 37 °C for 10 min, 37 °C for 15 min, room temperature RT for 5 min,10 min, and 15 min) were also evaluated.

#### 2.5.1. Determination of Cutoff Value

iELISA experiments were conducted according to the optimized conditions described above. The optimal cutoff value for the iELISA method was determined by measuring the OD450 value of 40 ASFV-negative sera. Based on statistical principles, the mean ($\bar{X}$) and standard deviation (SD) of the OD450 values were calculated; the cutoff value was then established using the following criteria: “negative value ≤ $\bar{X}$ + 2SD < suspicious value ≤ $\bar{X}$ + 3SD < positive value”.

#### 2.5.2. Specificity Analysis

According to the optimized conditions described above, the iELISA was performed to detect ASFV, PRRSV, CSFV, PCV2, PPV, FMDV, and PRV using standard positive pig-derived sera, as well as ASFV-negative serum. The OD450 values were measured for each sample using the established iELISA assays to evaluate the specificity of the method. This analysis was conducted to ensure that the iELISA is capable of specifically detecting ASFV antibodies without cross-reactivity with other common porcine pathogens, thereby confirming the assay’s specificity.

#### 2.5.3. Sensitivity Analysis

ASFV-positive sera were serially diluted from 1:100 to 1:12,800. The sensitivity of the pEP153R-iELISA assay was evaluated based on the previously determined cutoff value. This analysis was conducted to determine the lowest dilution at which the assay could reliably detect ASFV antibodies, ensuring the method’s sensitivity and reliability.

#### 2.5.4. Repeatability Analysis

The intra- and inter-assay repeatability of the established pEP153R-iELISA was evaluated using the optimized conditions described above. Six randomly selected positive and negative sera were tested on five replicates within the same batch and on five replicates from different batches. The mean ($\bar{X}$), standard deviation (SD), and coefficient of variation (CV) of the OD450 values were calculated for both sets of results using statistical analysis. This evaluation ensured the consistency and reliability of the assay across multiple runs and batches.

#### 2.5.5. Comparison with the Commercial Kit

A total of 276 swine clinical serum samples (from 5 farms with documented ASF/PRRS history, collected in 2024–2025) and RT-PCR confirmation criteria for ASFV positivity, consisting of 64 ASFV-positive sera and 212 ASFV-negative sera, were tested using a commercial ASF Competition detection kit (ID.VET, Montpellier, France). The same samples were analyzed using the two methods, and the coincidence rate of the two methods was calculated.

### 2.6. Rescue of Recombinant Virus

Initially, the cloned plasmid pPRRSV-EP153R was digested using the restriction enzyme *Swa* I (New England Biolabs, Beijing, China). The digestion product was then purified with QIAquick^®^ PCR Purification Kit (QIAGEN, Hilden, Germany) in strict accordance with the manufacturer’s instructions. Subsequently, the purified product was subjected to agarose gel electrophoresis for identification. Only the correctly linearized templates were advanced to the subsequent step. Following that, the linearized template underwent in vitro transcription according to the guidelines of the mMESSAGE mMACHINE T7 Transcription Kit (Invitrogen, Vilnius, Lithuania). After the transcription reaction was completed, in vitro transcripts were identified through RNA electrophoresis. Samples displaying the expected bands were selected for transfection into BHK-21 cells (Sunncell, Wuhan, China). The transfection process was carried out as detailed below: 1. BHK-21 cells were cultured in a six-well plate using DMEM medium supplemented with 10% FBS. The cells were harvested for use when they reached a confluence of 70–80%. 2. In a laminar flow hood, two EP tubes were prepared. Each tube was supplemented with 1 mL of Opti-MEM (Gibco, New York, NY, USA) and 5 μL of DMRIE-C Reagent (Invitrogen, Vilnius, Lithuania). Subsequently, 10 μL of the in vitro transcripts were added to each tube. After gentle mixing, the mixtures were slowly added to the BHK-21 cells. Simultaneously, a negative control without in vitro transcripts was set up. 3. The transfected cells were incubated at 37 °C in a humidified incubator for 8 h. After that, the medium was replaced with DMEM containing 2% FBS. 4. After continuous culturing for 72 h, the supernatant was collected and inoculated onto MARC-145 cells (Cytion, Eppelheim, Germany) that were in a good growth state and had a confluence of 70–80%. 5. The culture was maintained at 37 °C until the appearance of a cytopathic effect (CPE). The manifestation of CPE was regarded as an indication of the successful rescue of the recombinant virus. The collected supernatant was labeled as passage 1 (P1) and designated as rPRRSV-EP153R. Finally, the genetic stability of the recombinant virus was analyzed.

### 2.7. Virological Characteristics of Recombinant Virus

The recombinant virus rPRRSV-EP153R was serially diluted in a 10-fold gradient using DMEM supplemented with 2% FBS. Each dilution was then inoculated into 96-well cell culture plates seeded with MARC-145 cells, with eight replicates for each dilution. The plates were incubated at 37 °C in a 5% CO_2_ atmosphere for 5 days. After incubation, the CPE was observed and recorded, and the TCID_50_ was calculated using the Reed-Muench method. To observe and compare the plaque morphology differences between the recombinant virus and the parental virus, both the recombinant virus rPRRSV-EP153R and the parent virus vHuN4-F112 were diluted with DMEM and inoculated onto MARC-145 cells for 2 h. Following the adsorption period, a 2 × MEM culture medium containing 2% FBS and 2% low-melting-point agarose was added to the cells. After the agarose gel had solidified, the plates were inverted and placed in an incubator at 37 °C with 5% CO_2_. When the plaques had reached an appropriate size and shape, the cells were fixed with 4% paraformaldehyde and stained with gentian violet staining solution. Furthermore, MARC-145 cells were inoculated with rPRRSV-EP153R and vHuN4-F112 at a multiplicity of infection (MOI) of 0.1. The virus supernatants were collected at 12, 48, 60, 72, 84, 96, and 108 h post-infection (hpi). The titer of the collected virus supernatants was measured. The multi-step growth curves of the recombinant virus rPRRSV-EP153R and the parent virus were plotted by GraphPad Prism 10.1.2 software.

### 2.8. Verification of pEP153R Polyclonal Antibody

When the confluence of MARC-145 cells in cell culture plates reached 80–90%, the cells were infected with rPRRSV-EP153R and vHuN4-F112. The virus supernatants were diluted with DMEM, thoroughly mixed, and then added to the wells. A blank control group without virus infection was also set up. After 1 h of incubation, the medium was replaced with DMEM supplemented with 2% FBS, and the cells were cultured until the appearance of CPE. In addition, when the confluence of HEK-293T cells (Editgene, Guangzhou, China) in six-well plates reached 70–80%, the eukaryotic plasmid pCAGGS-EP153R was transfected into the cells using the lipo3000 transfection reagent. The transfection was allowed to proceed for 24 h. Following the culture period, the virus-infected MARC-145 cells and the plasmid-transfected HEK-293T cells were lysed separately. After centrifugation, the supernatants were collected for Western blotting to verify the efficacy of the prepared antibody. The pEP153R polyclonal serum, diluted at a ratio of 1:200, was used as the primary antibody, and goat anti-mouse IgG-HRP (Invitrogen, Vilnius, Lithuania), diluted at a ratio of 1:6000, was added as the secondary antibody. Meanwhile, ASFV-positive samples and purified proteins were also detected using the pEP153R antibody. The procedures of virus infection and plasmid transfection were as described above. After culture, the cells were fixed with ice-cold methanol for indirect immunofluorescence assay (IFA). The results were observed under an inverted fluorescence microscope, and images were captured for storage. Finally, the protein expression of pEP153R in the eukaryotic plasmid-transfected cells was visualized using laser confocal microscopy.

### 2.9. Safety Evaluation of the Recombinant Virus in Target Animals

The PRRSV vector, the parental virus vHuN4-F112, is an attenuated vaccine strain derived from the highly pathogenic PRRSV (HP-PRRSV) HuN4 strain through serial passaging and is well-documented for its virulence in piglets, possessing the absence of severe interstitial pneumonia [16,17]. Furthermore, all piglets were confirmed to be PRRSV- or ASFV-negative prior to the experiment via RT-PCR (nasal swabs and serum) and serology (IDEXX ELISA). The data did not show.

Ten 30-day-old piglets were randomly allocated into two experimental groups: the rPRRSV-EP153R group and the DMEM control group. Piglets in the immunization group were administered 2 mL of rPRRSV-EP153R solution at a concentration of 10^5^ TCID_50_/mL via intramuscular injection in the neck region, while the control group received 2 mL of DMEM. Throughout the experimental period, all animals were monitored daily for clinical signs, body weight changes, and rectal temperatures. Blood samples were collected at predetermined time points (0, 3, 7, 12, 14, 21, 28, and 35 dpi). Serum was separated and stored at −80 °C until use for antibody analysis. At 35 dpi, all piglets were humanely euthanized, and complete necropsy was performed. Gross pathological changes in major organs, including the heart, liver, spleen, lungs, kidneys, tonsils, and inguinal and mesenteric lymph nodes, were recorded, and tissue samples were subjected to histopathological examination. Furthermore, viral viremia or viral load in serum and tissue samples was quantified using reverse transcription quantitative PCR (RT-qPCR) to evaluate the safety profile of the recombinant virus in pigs. The ASFV pEP153R-specific antibodies were detected using the developed pEP153R iELISA, and the levels of PRRSV N protein-specific antibodies were evaluated using a commercial ELISA kit. Meanwhile, the changes in the levels of IL-4, IL-10, IFN-γ, and TNF-α cytokines in serum were monitored using the commercial ELISA kits (Cusabio, Wuhan, China) (Jonlnbio, Shanghai, China) (Beyotime, Shanghai, China).

### 2.10. Quantification and Statistical Analysis

Statistical analyses were performed using GraphPad Prism 10.1.2 software. Before applying parametric tests (Student’s *t*-test or two-way ANOVA with multiple comparison test for comparisons between immunized and control groups), normality of all datasets was verified using the Shapiro–Wilk test (*p* > 0.05), confirming normal distribution. Data are presented as means ± SD (*n* = 3 for in vitro experiments; *n* = 5 for in vivo pig experiments), and differences were considered significant at *p* < 0.05.

## 3. Results

### 3.1. Construction and Verification of Recombinant Plasmids Expressing ASFV pEP153R

**Figure 2:**
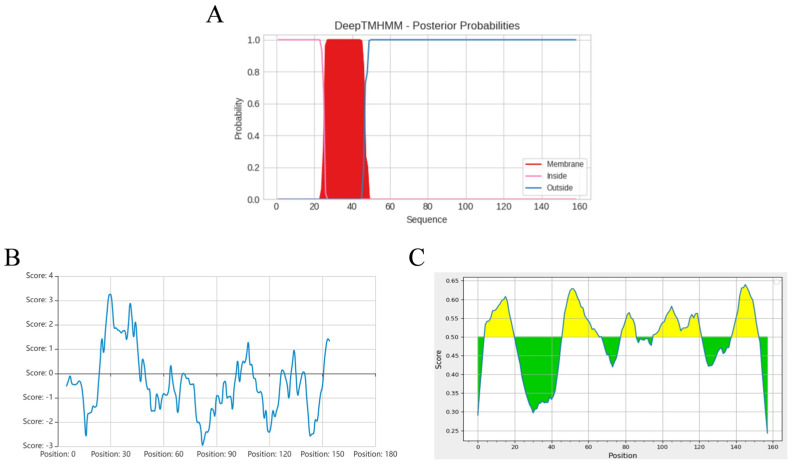
Bioinformatic analysis of pEP153R, including transmembrane region prediction (**A**), hydrophilicity profile (**B**), and B-cell epitope prediction with antigenic regions highlighted in yellow (**C**), performed using online tools detailed in the Methods. In (**A**), the *Y*-axis represents the probability scores; regions with values near 1 (red) indicate potential transmembrane domains, while pink and blue denote intracellular and extracellular regions, respectively. In (**C**), the yellow and green colors represent plus and minus scores, respectively, whereas the higher scores correspond to a greater likelihood of antigenic epitope.

**Figure 3:**
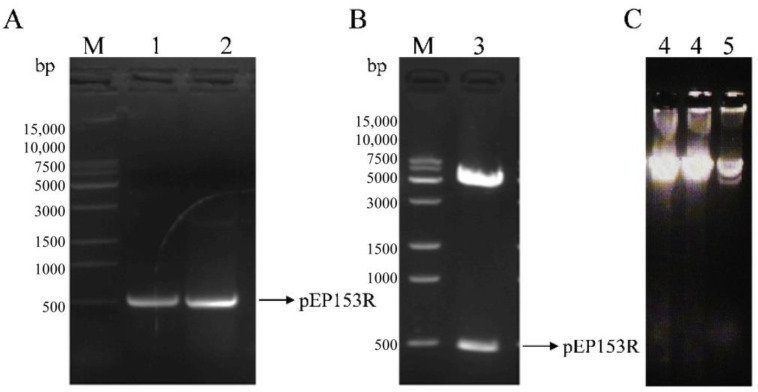
Identification of the prokaryotic plasmid and the infectious clone carrying *EP153R gene*. (**A**) PCR amplification of the *EP153R* coding sequence (M. 15 K DNA marker; 1, 2. *EP153R* amplicons). (**B**) Verification of the recombinant plasmid pCold-TF-EP153R (M. 15 K DNA marker; 3. pCold-TF-EP153R plasmid). (**C**) Verification of the recombinant plasmid pPRRSV-EP153R (4. pPRRSV-EP153R recombinant plasmid; 5. pHuN4-F112 positive plasmid).

Bioinformatics analysis revealed that the ASFV pEP153R is a transmembrane protein, with a predicted transmembrane domain spanning amino acid residues 26–46 (Figure 2A), consistent with the results of hydrophobicity profiling (Figure 2B). Predicted B-cell antigenic epitopes were identified in both the intracellular segment (1–25 aa) and the extracellular segment (47–153 aa), highlighted in yellow (Figure 2C). Based on these findings, the full-length pEP153R coding sequence was selected for cloning and subsequent fusion expression. The target *EP153R* fragment was amplified using homologous primers (F/R), yielding a product of approximately 501 bp, as confirmed by gel electrophoresis (Figure 3A). The recombinant prokaryotic plasmid pCold-TF-EP153R was constructed and verified by *Xho* I/*Eco*R I double digestion, producing fragments of approximately 5700 bp and 500 bp, in agreement with the expected sizes, and further confirmed by sequence analysis (Figure 3B). In addition, the recombinant plasmid pPRRSV-EP153R and the positive control plasmid pHuN4-F112 were verified by restriction enzyme analysis, revealing the expected sizes, with sequencing results confirming their correctness. Collectively, these findings confirmed the successful construction of the full-length infectious clone pPRRSV-EP153R (Figure 3C).

### 3.2. Expression, Purification, and Immunoreactivity of Recombinant ASFV pEP153R

**Figure 4:**
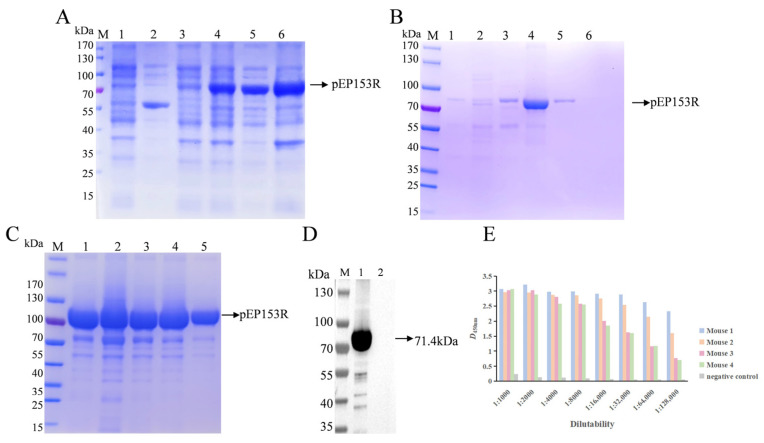
Identification of the expressed and purified products of ASFV pEP153R using SDS-PAGE and Western blotting. (**A**) SDS-PAGE analysis of pCold-TF-EP153R expression (M. protein marker; 1. No induction of vector; 2. Induction of vector; 3. No induction of pEP153R; 4. After induction of pEP153R; 5. Supernatant after induction and fragmentation of pEP153R; 6. Precipitation after induction and fragmentation of pEP153R). (**B**) SDS-PAGE analysis of purified pEP153R (M. protein marker; 1–6. Different concentrations of imidazole eluting buffers washed off-target proteins (5, 10, 20, 40, 60 and 80 mM). (**C**) SDS-PAGE analysis of concentration of pEP153R (M. protein marker; 1–5. 40 mM concentrations of imidazole eluting buffers washed off pEP153R). (**D**) Western blotting analysis of purified pEP153R (M. protein marker; 1. Recombinant pEP153R; 2. Vector protein). (**E**) Determination of antiserum titer by ELISA. The gray bar represents negative control. The blue, orange, pink, and green columns represent the antiserum of four individual mice. Data are presented as mean ± SD (*n* = 3).

The recombinant plasmid pCold-TF-EP153R was expressed following induction, and the predicted molecular mass of the target protein was approximately 71.4 kDa. SDS-PAGE analysis demonstrated that the pEP153R was present predominantly in the soluble fraction, with a band corresponding to the anticipated size (Figure 4A).

Purification of the recombinant pEP153R was achieved using Ni^2+^–nitrilotriacetic acid (Ni-NTA) affinity chromatography via its C-terminal His-tag. SDS-PAGE analysis revealed that the target protein of high purity (>90%) was effectively eluted with 40 mM imidazole (Figure 4B,C). Western blotting further confirmed that the purified His-tagged pEP153R was specifically recognized by an anti-His monoclonal antibody, indicating successful expression and preservation of antigenic properties (Figure 4D).

### 3.3. High Antigenicity of pEP153R Induces Robust Polyclonal Antibody Production

The concentration of the purified pEP153R was quantified and adjusted to 2 µg/mL, followed by coating onto ELISA plates at 4 °C overnight. Serial dilutions of mouse serum were prepared, and the antibody titer against pEP153R was determined via ELISA. Analysis of the OD450 values (Figure 4E) demonstrated that the endpoint titer was no less than 1:12,800. This high antibody titer indicates that the recombinant pEP153R possesses strong immunogenicity and can elicit a robust humoral immune response in mice, supporting its potential as a valuable immunogen for diagnostic and vaccine development.

### 3.4. Optimization and Validation of the pEP153R-Based Indirect iELISA for ASFV Antibody Detection

**Figure 5:**
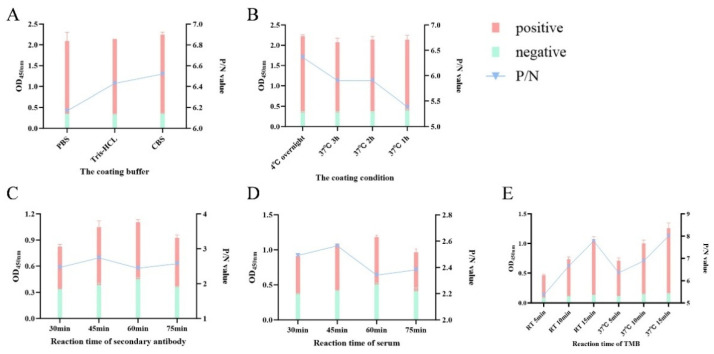
Establishment of pEP153R iELISA. Parameters tested include (**A**) optimal coating buffer, (**B**) optimal coating conditions, (**C**) optimal incubation time for enzyme-conjugated secondary antibody, (**D**) incubation time for ASFV serum, and (**E**) incubation time for TMB substrate.

Experimental optimization determined that the optimal antigen coating concentration for the pEP153R-based iELISA was 0.5 µg/mL, and the optimal serum dilution was 1:400 (Table 2). The low optimal antigen concentration and high serum dilution factor suggest that the established iELISA is highly sensitive and economical, reducing the consumption of reagents while maintaining excellent performance. The preferred coating condition involved using CBS (Figure 5A) at 4 °C overnight (Figure 5B). The optimal blocking condition was achieved with 5% (*w*/*v*) skim milk at 37 °C for 2 h, and the optimal second antibody dilution was 1:2000 (Table 3). Incubation at 37 °C for 45 min was optimal for both serum and secondary antibody (Figure 5C,D), while the optimal substrate (TMB) chromogenic reaction time was 15 min at 37 °C (Figure 5E).

**Figure 6:**
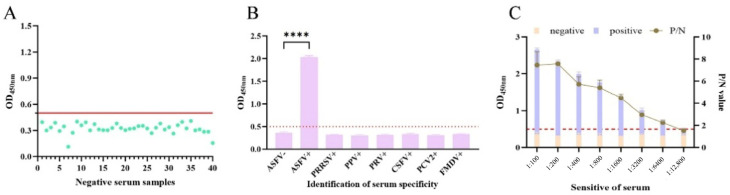
Characterization of the developed pEP153R iELISA. (**A**) The cutoff values of iELISA were determined using 40 sera from pigs free of ASFV. Based on the equations of ($\bar{X}$ + 2SD) and ($\bar{X}$ + 3SD), the OD450 > ($\bar{X}$ + 3SD) is considered as positive, the OD450 < ($\bar{X}$ + 2SD) is considered as negative, and the OD450 between is questionable. (**B**) The specificity of the pEP153R-iELISA was explored with 7 porcine pathogens, including ASFV, PRRSV, CSFV, PPV, PCV2, PRV and FMDV, plus a negative control (ASFV-) sample. **** *p* < 0.0001 by Student’s *t*-test. (**C**) The sensitivity of the iELISA was measured by using twofold serially diluted purified pEP153R and a control sample. Values are shown as mean ± SD (*n* = 3).

For assay cutoff determination, the mean OD450 value of 40 ASFV-negative sera was 0.323, with an SD of 0.059. Based on these values, results were interpreted as follows: negative value ≤ 0.440 < suspicious value ≤ 0.499 < positive value (Figure 6A). Accordingly, sera with OD450 values exceeding 0.499 were classified as positive.

Specificity testing revealed that all non-ASFV-positive sera produced OD450 values below the cutoff, except the ASFV standard positive serum (Figure 6B), confirming high assay specificity. Sensitivity assessment demonstrated that ASFV-positive serum remained above the cutoff value at dilutions up to 1:12,800 (Figure 6C).

Reproducibility evaluation using intra- and inter-assay variability showed that all coefficients of variation (CV) values were below 9% (Table 4), indicating excellent repeatability. To evaluate the performance and reliability of the newly developed pEP153R-iELISA against a commercial kit, 276 porcine clinical serum samples were collected. Both methods yielded identical results: 64 positive and 212 negative samples for the ELISA kit and 63 positive and 213 negative samples for pEP153R-iELISA (Table 5). The results showed that the coincidence rate of the two antibody detection kits was 98.18%. The 98.18% concordance with the commercial kit, coupled with high specificity and sensitivity, demonstrates that the pEP153R-based iELISA is a reliable serological tool for ASFV antibody detection, with potential for field application.

### 3.5. Generation and Genetic Stability of Recombinant PRRSV Expressing ASFV EP153R Gene

**Figure 7:**
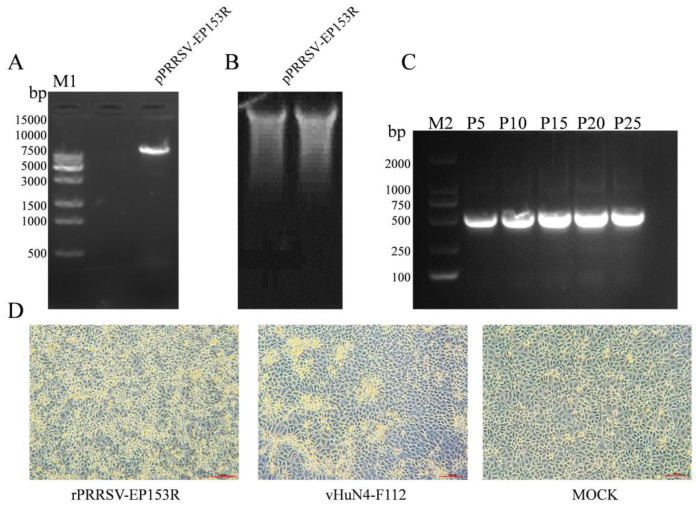
(**A**) Identification of recombinant plasmid by single enzyme digestion (M1. 15K DNA marker). (**B**) Validation of in vitro transcription RNA. (**C**) RT-PCR identification of rPRRSV-EP153R recombinant viruses (P5, P10, P15, P20 and P25 generations (M2. 2K DNA marker)). (**D**) Cytopathic effect (CPE) induced by the recombinant virus (20×).

The linearized template of the correctly sequenced pPRRSV-EP153R plasmid was verified by agarose gel electrophoresis, revealing a band of approximately 15 kb, consistent with the expected size (Figure 7A). The corresponding in vitro transcript was confirmed by RNA electrophoresis, which showed intact RNA without detectable degradation (Figure 7B). Extraction of viral RNA from the culture supernatants of rPRRSV-EP153R at passages P5, P10, P15, P20, and P25, followed by RT-PCR amplification, consistently detected the *EP153R* gene in all passages examined (Figure 7C). Following transfection of the in vitro transcript into BHK-21 cells and a 72 h incubation, supernatants were transferred to MARC-145 cells and cultured for an additional 3 days. Infected MARC-145 cells exhibited characteristic CPE, including cell aggregation and clustering morphology (Figure 7D), confirming the successful rescue of the recombinant virus rPRRSV-EP153R. Sequencing analysis further demonstrated that the *EP153R* gene insertion was stably maintained without mutations throughout viral rescue and serial passage (Figure S1).

### 3.6. Replication Kinetics and Plaque Morphology of rPRRSV-EP153R Compared with the Parental Strain

**Figure 8:**
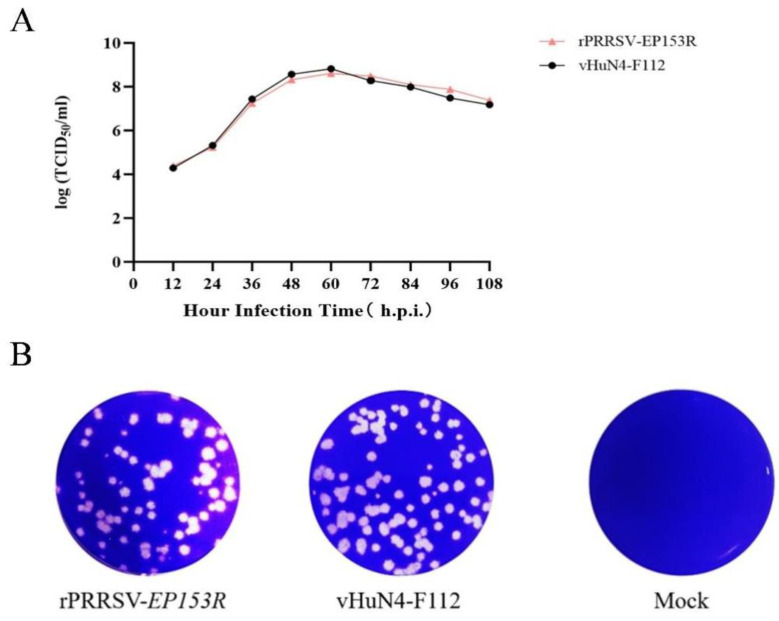
(**A**) Multi-step growth kinetics of recombinant virus rPRRSV-EP153R and parental virus vHuN4-F112. (**B**) Plaque morphology of rPRRSV-EP153R and vHuN4-F112. Viruses were serially diluted and inoculated onto MARC-145 cells. After adsorption, cells were overlaid with 2 × MEM containing 2% FBS and 2% low-melting-point agarose. Plaques were stained with crystal violet after 72 h. Data represent mean ± SD (*n* = 3).

The TCID_50_ of the recombinant virus rPRRSV-EP153R and its parental strain vHuN4-F112 was determined, revealing comparable infectious titers between the two viruses (Table 6). Multi-step growth curve analysis on MARC-145 cells showed that both viruses exhibited similar replication dynamics, reaching peak titer at 60 hpi; the difference was not statistically significant, indicating that the overall growth characteristics of the recombinant and parental viruses were analogous (Figure 8A). Plaque assays further demonstrated that the plaque morphology of rPRRSV-EP153R closely resembled that of vHuN4-F112, with only a marginal reduction in plaque numbers in the recombinant virus (Figure 8B). The minor plaque size variation between rPRRSV-EP153R and vHuN4-F112 is a phenotypic characteristic of PRRSV that may be driven by cell-specific replication rates rather than genetic differences. These findings suggest that insertion of the ASFV *EP153R* gene did not significantly alter the replication efficiency or cytopathic characteristics of the recombinant PRRSV in MARC-145 cells. In conclusion, the insertion of the ASFV *EP153R* gene into the PRRSV genome did not impair viral replication fitness, which is a critical prerequisite for the development of a stable live vectored vaccine.

### 3.7. Specific Detection and Subcellular Localization of ASFV pEP153R Expressed by rPRRSV-EP153R

**Figure 9:**
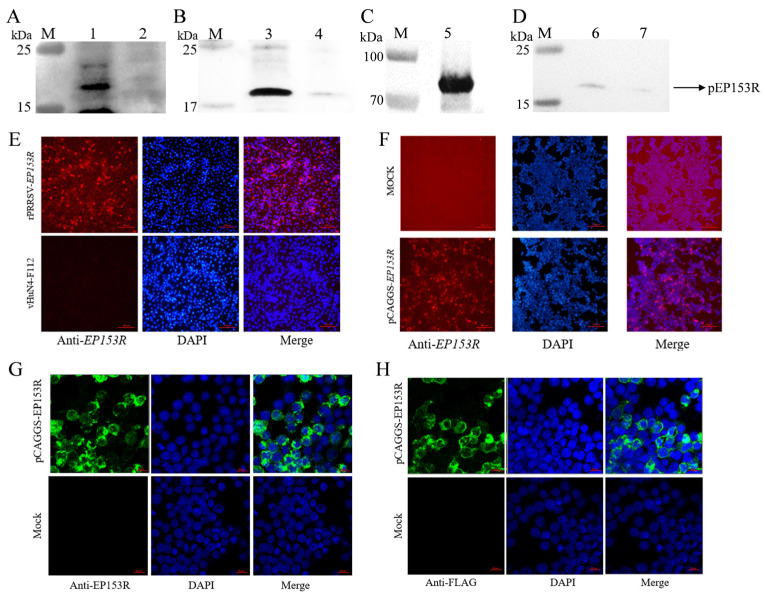
Detection of pEP153R expression in various samples via Western blotting (WB) and indirect immunofluorescence assay (IFA): (**A**) recombinant virus, (**B**) eukaryotic plasmid, (**C**) recombinant protein and (**D**) ASFV-protein sample (M. protein marker; 1. rPRRSV-EP153R; 2. vHuN4-F112; 3. pCAGGS-EP153R; 4. pCAGGS empty vector; 5. purified pEP153R; 6. ASFV-positive sample; 7. ASFV-negative sample). (**E**,**F**) Indirect immunofluorescence assay (IFA) of pEP153R expression in rPRRSV-EP153R or pCAGGS-EP153R was performed using anti-pEP153R polyclonal antibody (20×). Cells were fixed with ice methanol, blocked with 5% BSA, and incubated with polyclonal serum (1:200) and goat anti-mouse fluorescent secondary antibody IgG-594 (1:2000). Nuclei were stained with DAPI (Blue fluorescence). Red fluorescence signal indicates pEP153R expression. (**G**,**H**) Confocal microscopy imaging of pEP153R-antibody binding in transfected MARC-145 cells (20×). After eukaryotic transfection for 24 h, cells were permeabilized, blocked, incubated with anti-pEP153R (1:200) or anti-Flag (1:2000) antibody, followed by fluorescent secondary antibody (1:2000), and fixed on the slide with a sealer. Nuclei were stained with DAPI (Blue fluorescence). Green fluorescence indicates pEP153R expression.

MARC-145 cells were infected with rPRRSV-EP153R and the vHuN4-F112 strain, respectively. Viral proteins were subsequently harvested and subjected to Western blotting analysis. The results demonstrated that the pEP153R antibody specifically recognized the foreign protein expressed by rPRRSV-EP153R, as evidenced by the presence of distinct target bands. Notably, no reactivity was observed with the vHuN4-F112 strain (Figure 9A). In parallel, 293 T cells were transfected with 2.0 μg of the eukaryotic expression plasmid pCAGGS-EP153R, and the expressed protein was collected for Western blotting analysis. The pEP153R polyclonal antibody exhibited specific recognition of the exogenous pEP153R expressed by the eukaryotic plasmids (Figure 9B). Furthermore, Western blotting assays confirmed that the antibody displayed strong specificity against both the ASFV-positive sample and the purified recombinant pEP153R (Figure 9C,D).

To further corroborate the Western blotting findings, IFA was conducted using rPRRSV-EP153R and pCAGGS-EP153R. No red fluorescence was detected in the control group, whereas positive signals were observed in cells infected with rPRRSV-EP153R using the in-house prepared pEP153R antibody (Figure 9E). Similarly, red fluorescence was detected in cells transfected with the eukaryotic expression plasmid pCAGGS-EP153R (Figure 9F). These results collectively indicate that the pEP153R antibody generated in our laboratory exhibits high specificity. Additionally, the ASFV *EP153R* gene product was stably expressed by the recombinant rPRRSV-EP153R virus constructed and rescued in our laboratory. To determine the subcellular localization of pEP153R, MARC-145 cells were infected with the recombinant virus for 48 h, followed by confocal laser microscopy using the pEP153R antibody. The results revealed that pEP153R specifically interacted with viral proteins, and green fluorescence signals were predominantly localized at the cell membrane of MARC-145 cells, suggesting that pEP153R is membrane-associated (Figure 9G,H). These findings underscore the specificity and utility of the pEP153R antibody in detecting and localizing the pEP153R, providing valuable insights into its role in viral pathogenesis. In short, the specific recognition of pEP153R expressed in different systems confirms the high quality and utility of the prepared polyclonal antibody for subsequent diagnostic and functional studies and simultaneously verifies the correct expression of the target antigen by the recombinant virus.

### 3.8. Assessment of Safety and Induction of PRRSV and ASFV-Specific Antibody Responses by rPRRSV-EP153R in Piglets

**Figure 10:**
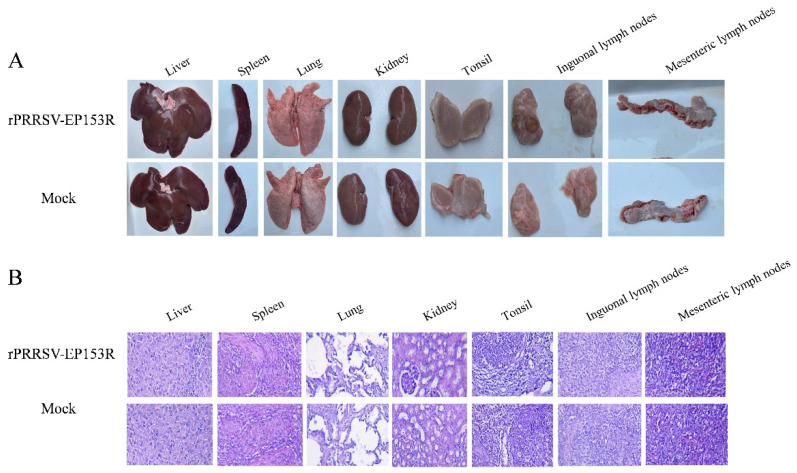

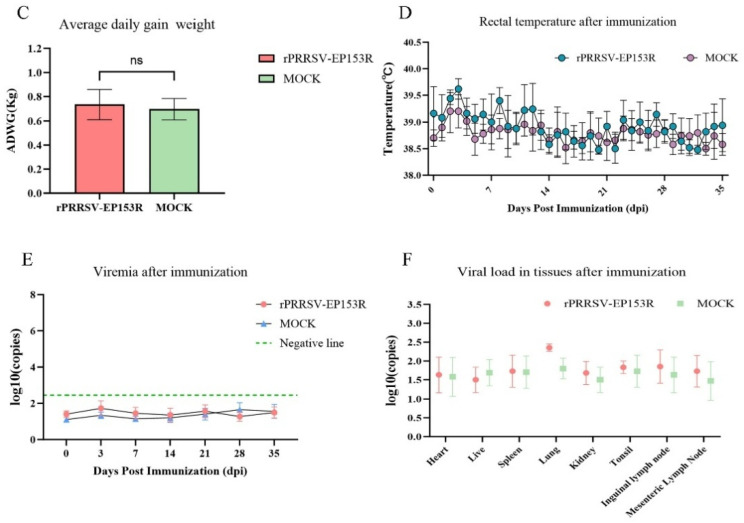
Safety assessment of pigs with rPRRSV-EP153R immunization. (**A**) Macroscopic observation of major organs and tissues following immunization. (**B**) Histopathological analysis of tissue sections from immunized pigs. (**C**) Evaluation of average daily weight gain after immunization between rPRRSV-EP153R and DMEM. Statistical analysis was performed by Student’s *t*-test. ns, no significant differences (*p* > 0.05). (**D**) Daily rectal temperature records of piglets after immunization. (**E**) Detection of viral RNA in serum samples and (**F**) viral load in tissues to determine rPRRSV-EP153R immunological efficacy.

We assessed the safety profile of the recombinant live virus vector vaccine rPRRSV-EP153R in target pigs. Following the established immunization protocol, piglets were intramuscularly administered with 2 mL of rPRRSV-EP153R virus solution at a concentration of 10^5^ TCID_50_/mL. Throughout the experimental period, no overt clinical signs or adverse reactions were detected in any of the vaccinated animals. At 35 dpi, gross pathological examination and histopathological analysis were performed on eight major organs or tissues, including the heart, liver, spleen, lung, kidney, tonsil, inguinal lymph node, and mesenteric lymph node. Necropsy results revealed no macroscopic or microscopic abnormalities in either the vaccinated group or the control group (Figure 10A,B). Furthermore, the vaccinated piglets exhibited normal average daily weight gain, with no evidence of growth retardation or fever throughout the observation period (Figure 10C,D). No viremia, viral shedding, or detectable viral load was observed in the rPRRSV-EP153R group during the study, which was consistent with the findings in the DMEM blank control group (Figure 10E,F). Collectively, these results demonstrate that the absence of clinical signs or pathological lesions confirms the safety of the rPRRSV-EP153R in its natural host. The concurrent induction of specific antibodies against both PRRSV and ASFV antigens underscores the dual functionality of this recombinant virus.

**Figure 11:**
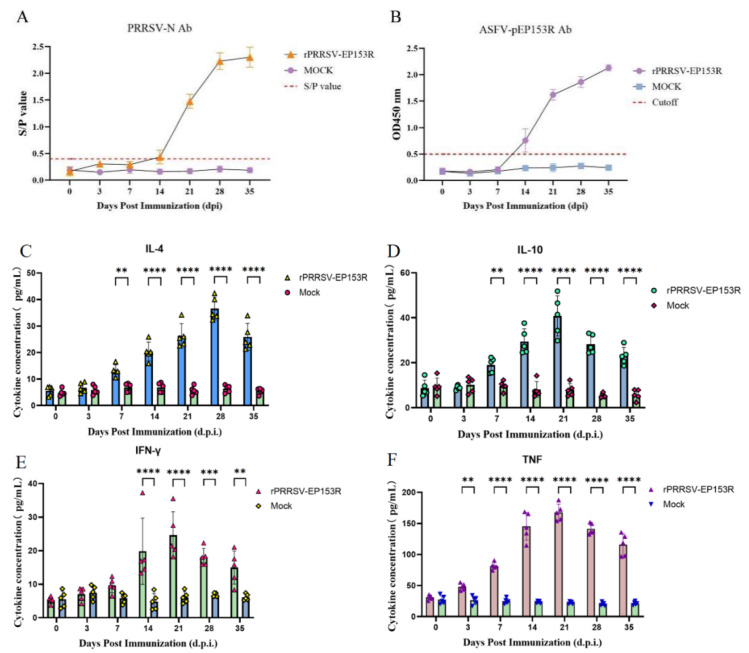
Serological and cytokine evaluation of pigs immunized with rPRRSV-EP153R. (**A**) PRRSV-N-specific antibody S/P ratios (detected via IDEXX PRRS ELISA kit) in serum samples from rPRRSV-EP153R-immunized pigs and DMEM control pigs at the indicated days post-immunization (dpi). (**B**) ASFV-pEP153R-specific antibody OD450 values (detected via optimized pEP153R-iELISA) in the two groups over time. (**C**–**F**) Dynamic changes in serum cytokine levels (IL-4, IL-10, IFN-γ, and TNF-α, respectively; detected via JONLNBIO ELISA kit) in immunized and control pigs. Statistical analyses were performed using two-way ANOVA with multiple comparison tests to assess differences between groups at each time point. Prior to analysis, data normality was verified using the Shapiro–Wilk test (*p* > 0.05 for all datasets), confirming suitability for parametric testing. ** *p* < 0.01, *** *p* < 0.001, **** *p* < 0.0001. Data are presented as mean ± SD (*n* = 5 per group).

All experiment pigs were 30-day-old, PRRSV/ASFV-negative (verified by RT-PCR and IDEXX ELISA), and housed under identical conditions. The immunization protocol was strictly standardized (2 mL 10^5^ TCID_50_/mL rPRRSV-EP153R via intramuscular injection). The humoral immune response against PRRSV N-specific antibodies in vaccinated piglets was monitored at the predetermined time point, using a commercial ELISA kit. A significant increase in PRRSV N-specific antibody titer was observed by 21 dpi (Figure 11A). Notably, porcine immune serum (35 dpi) was serially diluted to detect endpoint titer via the established pEP153R-iELISA. All pigs in the rPRRSV-EP153R immunized group produced pEP153R-specific antibodies, with detectable anti-pEP153R titers (up to 1:1600); though relatively low, these titers aligned with previously reported humoral responses of other ASFV subunit vaccines. In contrast, all pigs in the DMEM group had a serum titer <1:100. Collectively, these results confirm that rPRRSV-EP153R induces a specific, detectable humoral immune response against the ASFV pEP153R in piglets (Figure 11B and Figure S2). Low variability in antibody titers among the five immunized pigs (1:1600–1:3200) reflects the reproducibility of the antigen delivery and immunogenicity, a key advantage for potential clinical application in swine herds.

Additionally, changes in the levels of cytokines (IL-4, IL-10, IFN-γ, and TNF-α) in serum were monitored. The levels of IL-4 and IL-10 in the immunized group showed significant differences compared to the blank control group at 7 dpi, with IL-10 reaching its peak faster than IL-4; IFN-γ was produced more slowly compared to IL-4 and IL-10, showing significant differences between the immunized group and the blank control group only at 14 dpi. TNF-α cytokine in the immunized group showed significant differences compared to the blank control group at the production level only at 3 dpi. The above results indicate that the recombinant vaccine rPRRSV-EP153R can effectively stimulate the consistent and detectable humoral and cellular immune responses in pigs (Figure 11C–F).

## 4. Discussion

Since the initial outbreak of ASF in China in 2018, the disease has rapidly disseminated across multiple provinces and municipalities, establishing a stable endemic presence and inflicting substantial economic losses on the domestic swine industry. Viral infectious diseases are typically amenable to effective prevention through vaccination. However, the development of a safe and efficacious commercial ASF vaccine has been impeded by several factors, including the large genome of ASFV, the encoding of numerous proteins with undefined functions, a complex immune evasion mechanism, and inadequate cross-protective efficacy. In the realm of ASF vaccine research and development, several candidate antigenic proteins, such as ASFV p30, p54, p72, and CD2v, have demonstrated significant research potential. Nonetheless, the majority of ASFV protective antigens utilized in current subunit vaccines fail to confer complete protection or elicit a robust cellular immune response [18]. Consequently, there is an imperative need to identify highly protective antigenic targets and efficient antigen expression vectors to develop effective vaccine strategies and diagnostic methods for prevention and control of ASF. The pEP153R, a C-type lectin encoded by the ASFV, features N-glycosylation and phosphorylation sites and is detectable during both the early and late stages of viral infection, making it a promising candidate as a potential antigenic target for ASFV. Studies have shown that the deletion of the CD2v and *EP153R* genes from the ASFV Benin Δ*9GL* strain significantly impaired the in vitro replication capacity of the recombinant virus, which was not observed with the deletion of the *9GL* gene alone, thereby diminishing its viability as an experimental vaccine candidate [19]. Further research demonstrated that the removal of the *EP402R* gene from the ASFV Benin Δ*DP148R* strain markedly reduced viral persistence in the bloodstream. Building on this, the additional deletion of the *EP153R* gene (resulting in the Benin Δ*DP148R*Δ*EP402R*Δ*EP153R* strain) further attenuated the virus, suggesting a synergistic effect between the *EP153R* and *EP402R* genes in reducing viral virulence at the systemic level [20]. While multi-gene deletion strategies may enhance the safety of recombinant vaccines, they can also compromise immunogenicity. Currently, ASFV subunit vaccines remain in the laboratory research phase and lag live attenuated vaccines in development. Notably, progress has been made in expressing ASFV foreign genes using viral vectors. For example, Goatley et al. (2020) cloned eight virulence genes of ASFV (*B602L*, p72, p30, p54, *E199L*, *EP153R*, *F317L* and *MGF505*-*5R*) into adenovirus vectors for primary immunization in pigs, followed by boosting the same genes expressed in the Modified Vaccinia Virus Ankara (MVA) vector. Subsequent challenge with ASFV resulted in 100% protective efficacy [21]. It has been established that pEP153R is a transmembrane protein, and consequently, anti-pEP153R antibodies are not expected to exhibit neutralizing activity or effectively inhibit viral replication. Nevertheless, due to stringent biosafety requirements associated with handling live African swine fever virus (ASFV), which necessitate BSL-3 or higher containment, this study was unable to perform live-virus neutralization assays using the generated pEP153R antisera. To address this limitation in future work, we plan to employ a pseudovirus-based neutralization assay as an alternative approach. Importantly, we emphasize that antibodies directed against membrane-associated antigens such as pEP153R may still contribute to protective immunity through Fc-mediated effector mechanisms, including antibody-dependent cellular cytotoxicity (ADCC), which can facilitate the clearance of infected cells. Given these potential immunomodulatory roles, further investigation of pEP153R in the context of ASFV infection is both justified and essential. In brief, these findings underscore the critical role of ASFV antigenic proteins in vaccine development and highlight the need for further investigation into the functional mechanisms of these key antigens to inform the effective ASF vaccine design.

In addition to the ongoing prevalence of ASFV in China, there has also been a notable upward trend in the PRRS. This situation underscores the urgent need to develop effective vaccines capable of providing protection against both ASFV and PRRSV infections. With the advancement of reverse genetic technology, research on using the attenuated strain of PRRSV as a live viral vector for the expression of foreign viral proteins has reached a certain level of maturity. For example, Xu et al. successfully rescued a recombinant PRRSV that could stably express a partially dominant B-cell gene fragment. The insertion site of this foreign gene was in the non-structural protein 2 (Nsp2) region [22]. When pigs were immunized with this recombinant virus, it not only elicited anti-PRRSV antibodies but also anti-Newcastle Disease Virus antibodies. Li et al. rescued a recombinant PRRSV that could stably incorporate the TRS6 and interleukin 4. The insertion site was situated between ORF7 and the 3′ UTR [23]. After immunizing pigs with this recombinant virus, an increase in the number of CD4^+^ and CD8^+^ T cells and the level of interleukin 4 was observed. Moreover, in our laboratory, GAO et al. selected the natural insertion site between ORF1b and ORF2a of PRRSV to rescue the recombinant virus rPRRSV-E2. This recombinant virus could stably express the E2 protein of the CSFV. Pigs immunized with the recombinant virus demonstrated simultaneous resistance to the challenges of highly pathogenic PRRSV and CSFV [15]. This makes it one of the most promising vaccine candidates. After presenting all the above information, the pEP153R exhibits promising application potential in the development of ASF vaccines. The recombinant PRRSV vector carrying this gene provides a feasible strategy for the development of bivalent vaccines against ASF and PRRS. Therefore, to develop a novel genetically engineered live vaccine for the simultaneous prevention and control of ASFV and PRRSV, after recognizing the significance of ASFV pEP153R and the maturity of PRRSV live virus vector technology, we constructed the recombinant virus rPRRSV-EP153R. Figure 7 and Figure 8 illustrate that the successful insertion of the *EP153R* gene between ORF1b and ORF2a did not impact the replication capacity of PRRSV, further validating the feasibility of this vector system.

We have developed an iELISA method targeting the ASFV pEP153R protein, a viral antigen expressed during both early and late stages of infection and harboring abundant T-cell epitopes. This dual-expression profile enables the assay to serve not only for the diagnosis of natural ASF infections but also for monitoring antibody responses following immunization with recombinant vaccines such as rPRRSV-EP153R; therefore, the method provides a technical foundation for integrated “detection–immunization” strategies in ASF control. The optimal conditions were determined as follows: antigen coating concentration at 0.5 μg/mL and serum dilution at 1:400. The low antigen requirement reduces assay costs, while the high serum dilution minimizes interference from non-specific serum components. Additional optimizations, including overnight coating at 4 °C and blocking with 5% skim milk at 37 °C for 2 h, enhanced detection efficiency without compromising performance, enabling rapid processing of large-scale samples. Specificity was confirmed by testing positive sera against seven common porcine pathogens, including PRRSV and CSFV; only ASFV-positive sera yielded OD450 values above the cutoff, with no cross-reactivity observed, thereby ensuring accurate and specific identification of ASFV infection and minimizing false-positive diagnoses. Notably, the assay demonstrated exceptional sensitivity, successfully detecting ASFV-positive sera diluted up to 1:12,800, well beyond the typical upper limit of conventional serological assays. This high sensitivity allows reliable detection of low-titer antibody samples, such as those from early infection or the initial phase post-vaccination, reducing the risk of false-negative results and making the method particularly valuable for early surveillance and epidemiological investigations. The method exhibited excellent reproducibility, with intra- and inter-batch CVs below 9%, meeting stringent quality control criteria for serological assays. This consistency across batches, operators, and time points supports its robustness and broad applicability in multi-laboratory settings. Furthermore, a comparative analysis using 276 clinical serum samples showed a 98.18% concordance with ELISA kit, validating the reliability of our method and positioning it as a credible alternative or complementary tool to existing commercial kits. The current assay relies on mouse-derived anti-pEP153R polyclonal antibodies, which offer advantages in cost-effectiveness and broad epitope recognition. However, their specificity is inherently lower than that of monoclonal antibodies. Future refinement through the development of monoclonal antibody-based reagents could further reduce non-specific binding and enhance analytical precision. In summary, the pEP153R-iELISA method demonstrates outstanding performance in specificity, sensitivity, stability, and cost-efficiency, offering a reliable platform for both serodiagnosis and vaccine immune monitoring in ASF control. Its systematic optimization framework and validation approach may serve as a model for developing serological assays for other animal viral diseases. With future enhancements in antibody specificity and multimodal integration, this method holds strong potential to advance precision prevention and control of ASF.

Following immunization, the serum antibody titer attained a level of 1:1600 at 35 days post-infection (dpi). Furthermore, we monitored the changes in specific antibody levels (PRRSV N and ASFV pEP153R) and cytokine levels (IL-4, IL-10, IFN-γ, and TNF-α) in the serum of immunized piglets. Serum cytokine detection, while limited in directly measuring T-cell specificity, still reflects coordinated immune activation, supported by the temporal alignment of IFN-γ elevation with pEP153R-specific antibody production. Meanwhile, the findings of cellular immune response demonstrate that rPRRSV-EP153R not only induces a humoral response but also activates a specific, consistent, and detectable cellular immune response, characterized by the production of key cytokines associated with T-helper cell responses. While the antibody titer targeting NSPs may exhibit a lower magnitude compared to that directed against viral structure proteins, the live vector-based delivery platform employed in the present study demonstrated efficacy in antigen delivery and the induction of a potent antigen-specific immune response, and the concurrent induction of Th1/Th2 balanced cellular immunity (evidenced by elevated IFN-γ, IL-4, and IL-10) supports the vaccine’s potential to elicit comprehensive immune protection—consistent with the fact that ASFV clearance relies on both humoral and cellular immunity. This balanced immune activation is also crucial for effective vaccine-induced protection against viral infections. Therefore, this finding not only validates the feasibility of the selected vector system but also establishes a solid experimental basis for the subsequent advancement of related immunological research and product development.

However, in this study, the small sample size, like five mice for antibody preparation and five piglets for immunization, may reduce the statistical power of the results, particularly in assessing individual immune response variations. Therefore, this is merely a preliminary verification result. Moreover, the study primarily focused on immune responses, while the evaluations of CD4^+^ and CD8^+^ T cells were insufficient, requiring further investigation. Compared to other ASFV vaccine candidates, such as adenovirus- or MVA-vectored platforms expressing multiple ASFV antigens [24,25,26], the present study employs a PRRSV-based live vector delivering *EP153R* gene, offering distinct advantages. As a natural porcine pathogen, PRRSV exhibits tropism for porcine macrophages, the primary target cells of ASFV, potentially enhancing antigen presentation and ASFV-specific immunity. Moreover, the attenuated PRRSV backbone enables dual immunization against both PRRSV and ASFV, constituting a bivalent strategy that could streamline vaccination protocols and lower costs. Live vectored vaccines like this typically induce a detectable immune response—cellular immunity, crucial for controlling ASFV—compared to subunit or inactivated formulations. While multi-antigen ASFV vaccines have demonstrated efficacy [21], the monovalent pEP153R approach minimizes potential immune interference and facilitates clearer identification of protective correlates. To our knowledge, this is the first PRRSV-vectored ASFV vaccine expressing pEP153R, while other bivalent vaccines have been developed using similar strategies [16], representing a novel strategy against two major swine diseases simultaneously. The rPRRSV-EP153R immunization experiments induced cellular and humoral immune responses that could recognize the whole virus. However, no clear correlation of protection emerged that could predict the clinical outcomes after challenge with virulent ASFV. Thus, subsequent studies will include challenge trials to assess protective efficacy.

## 5. Conclusions

Based on these characteristics, a highly specific, sensitive, and reproducible iELISA targeting the pEP153R was developed and optimized. Although currently in the preliminary validation phase, this methodology established a robust platform for subsequent antigenic characterization studies and could significantly contribute to timely ASF diagnosis and outbreak containment strategies. Furthermore, we successfully generated the recombinant virus rPRRSV-EP153R, which stably expresses the ASFV *EP153R* coding protein. Comprehensive comparative analyses of viral replication kinetics and plaque morphology between rPRRSV-EP153R and the parental strain vHuN4-F112 revealed no statistically significant differences, suggesting the genetic modification did not impair viral fitness. Immunization trials with rPRRSV-EP153R elicited a specific, consistent, and detectable immune response and specific humoral immune responses against both PRRSV and ASFV antigens, indicating its potential as a promising live vector vaccine candidate.

In conclusion, these findings suggest that utilizing PRRSV live vectors to express ASFV heterologous genes for the development of recombinant virus vaccine strains represents a significant advancement in veterinary vaccinology. This innovative approach is expected to play a pivotal role in the future development of comprehensive prevention and control strategies against porcine infectious diseases, particularly in the context of ASF management. Subsequent research endeavors should prioritize long-term protection studies to assess the persistence of specific antibody responses against ASFV and PRRSV elicited by the recombinant virus. Significantly, we intend to evaluate this vaccine candidate under real farm conditions, mimicking the intricate epidemiological milieu of commercial pig farms, which often involve the co-circulation of multiple pathogens. This approach aims to comprehensively evaluate its adaptability, immunological efficacy, and potential safety within practical production settings, thereby laying a more robust foundation for its eventual implementation in the prevention and control of ASFV and PRRSV in the swine industry.

## Figures and Tables

**Table 1:** The primers used for this paper.

Primer Name	Sequence of Primer (5′-3′)
pCOLD-pEP153R-F	5′-GCTCGGTACCCTCGAGATGTTCTCTAACAAAAAATATATTGGACTGATCAACAAGA-3′
pCOLD-pEP153R-R	5′-CGACAAGCTTGAATTCTCACTTATCGTCGTCATCCTTGTAATCT-3′
pCAGGS-pEP153R-F	5′-CATCATTTTGGCAAAGAATTCATGTTCTCTAACAAAAAATATATTGGACTG-3′
pCAGGS-pEP153R-R	5′-GAGGGAAAAAGATCCGATATCTCACTTATCGTCGTCATCCTTGTAA-3′
pEP153R-F	5′-TCATACATCCGAGTTCCTGTT-3′
pEP153R-R	5′-GAAATATTGTCATGGCGAGGC-3′

**Table 2:** Determination of optimal antigen coating concentration and serum dilutions.

The Dilution Degree of ASFV-Positive Serum	OD450	The Concentration of Antigen Coating (µg/mL)					
		0.5	1	2	4	8	10
1:40	P	2.215	1.855	1.617	1.646	1.619	1.467
	N	0.511	0.324	0.257	0.263	0.239	0.247
	P/N	4.335	5.725	6.292	6.259	6.774	5.939
1:80	P	2.627	2.135	1.757	1.777	1.688	1.784
	N	0.419	0.381	0.283	0.288	0.247	0.275
	P/N	6.270	5.604	6.208	6.170	6.834	6.487
1:100	P	2.435	2.115	1.753	1.793	1.933	1.760
	N	0.351	0.347	0.308	0.298	0.288	0.278
	P/N	6.937	6.095	5.692	6.017	6.712	6.331
1:200	P	2.850	2.256	2.010	2.041	1.975	1.874
	N	0.367	0.319	0.308	0.323	0.312	0.388
	P/N	7.766	7.072	6.526	6.319	2.313	4.830
1:400	P	2.534	2.371	2.309	2.337	2.313	2.266
	N	0.319	0.303	0.412	0.425	0.393	0.520
	P/N	7.944	7.825	5.604	5.499	5.885	4.358

P: OD450 of pigs’ positive samples; N: OD450 of pigs’ negative samples.

**Table 3:** Determination of optimal blocking conditions and second antibody dilutions.

Different Blocking Buffer	OD450	The Blocking Time											
		37 °C 1 h				37 °C 2 h				37 °C 3 h			
		The Dilution Degree of Enzyme-Labeled Secondary Antibody											
		1:2000	1:4000	1:8000	1:10,000	1:2000	1:4000	1:8000	1:10,000	1:2000	1:4000	1:8000	1:10,000
5% skim milk	P	2.766	2.752	2.128	1.421	2.782	2.496	2.019	1.235	2.397	2.086	1.584	0.970
	N	0.365	0.342	0.223	0.176	0.379	0.257	0.201	0.116	0.223	0.200	0.204	0.103
	P/N	7.58	8.05	9.54	8.07	7.34	9.71	10.04	10.65	10.75	10.43	7.76	9.420
2% skim milk	P	2.434	2.116	1.61	1.031	2.125	1.903	1.390	0.934	1.733	1.587	1.084	0.722
	N	0.258	0.275	0.250	0.121	0.272	0.175	0.163	0.095	0.166	0.177	0.149	0.095
	P/N	9.43	7.69	6.44	8.52	7.81	10.87	8.53	9.83	10.44	8.97	7.28	7.60
5% BSA	P	2.051	1.768	1.388	0.737	1.731	1.393	1.066	0.387	1.578	1.411	1.075	0.607
	N	0.172	0.141	0.112	0.097	0.133	0.111	0.107	0.074	0.123	0.111	0.093	0.081
	P/N	11.92	12.54	12.39	7.60	13.02	12.55	9.96	5.23	12.83	12.71	11.56	7.49
2% BSA	P	1.938	1.398	0.136	0.107	1.366	0.947	0.852	0.491	1.176	0.98	0.863	0.476
	N	0.900	0.754	0.208	0.084	0.115	0.093	0.184	0.072	0.111	0.099	0.091	0.071
	P/N	2.15	1.85	0.65	1.27	11.88	10.18	4.63	6.82	10.59	9.90	9.48	6.70

P: OD450 of pigs’ positive samples; N: OD450 of pigs’ negative samples.

**Table 4:** The reproducibility test of pEP153R-iELISA.

Sample No.		Intra-Assay Cv (%)		Inter-Assay Cv (%)	
		$\bar{X}$ ± SD	Cv (%)	$\bar{X}$	Cv (%)
Positive samples	1	0.706 ± 0.052	7.303	0.651 ± 0.043	6.602
	2	0.564 ± 0.021	3.759	0.528 ± 0.046	8.736
	3	0.702 ± 0.034	4.861	0.696 ± 0.047	6.745
Negative samples	4	0.106 ± 0.007	6.212	0.100 ± 0.008	8.141
	5	0.105 ± 0.005	4.762	0.122 ± 0.008	6.296
	6	0.116 ± 0.005	4.100	0.109 ± 0.008	7.355

**Table 5:** The coincidence rate test of pEP153R-iELISA.

Methods	Commercial Detection Kit				Coincidence Rate
		Positive	Negative	Total	
**pEP153R-iELISA**	Positive	61	2	63	
	Negative	3	210	213	98.18%
	Total	64	212	276	

**Table 6:** TCID_50_ of recombinant virus.

Viruses	TCID_50_/mL
rPRRSV-EP153R	5.87 × 10^7^
vHuN4-F112	8.16 × 10^7^

## Data Availability

The authors confirm that the data supporting the findings of this study are available within the article and its Supplementary Materials.

## Supplementary Materials

The following supporting information can be downloaded at https://www.mdpi.com/article/10.3390/vaccines13111110/s1, Figure S1: The sequencing results comparison of rPRRSV-EP153R including P5, P10, P10, P20 and P25 generations; Figure S2: Detection of the endpoint titer of the pig serum antibodies after 35 days post-immunization. Dashed horizontal red line represents the pEP153R-iELISA positive cutoff (OD_450_ = 0.499).
