# Supplementary material for: Expression and Immunological Characterization of African Swine Fever Virus EP153R Protein for Serodiagnosis and Its Delivery via a Recombinant PRRSV Live Vector
Source: Vaccines (Basel). 2025 Oct 29;13(11):1110. doi: 10.3390/vaccines13111110 (PMC12656761; doi:10.3390/vaccines13111110)
Supplement: Supplementary file 1 [file vaccines-13-01110-s001.zip › vaccines-3922403-supplementary.pdf]

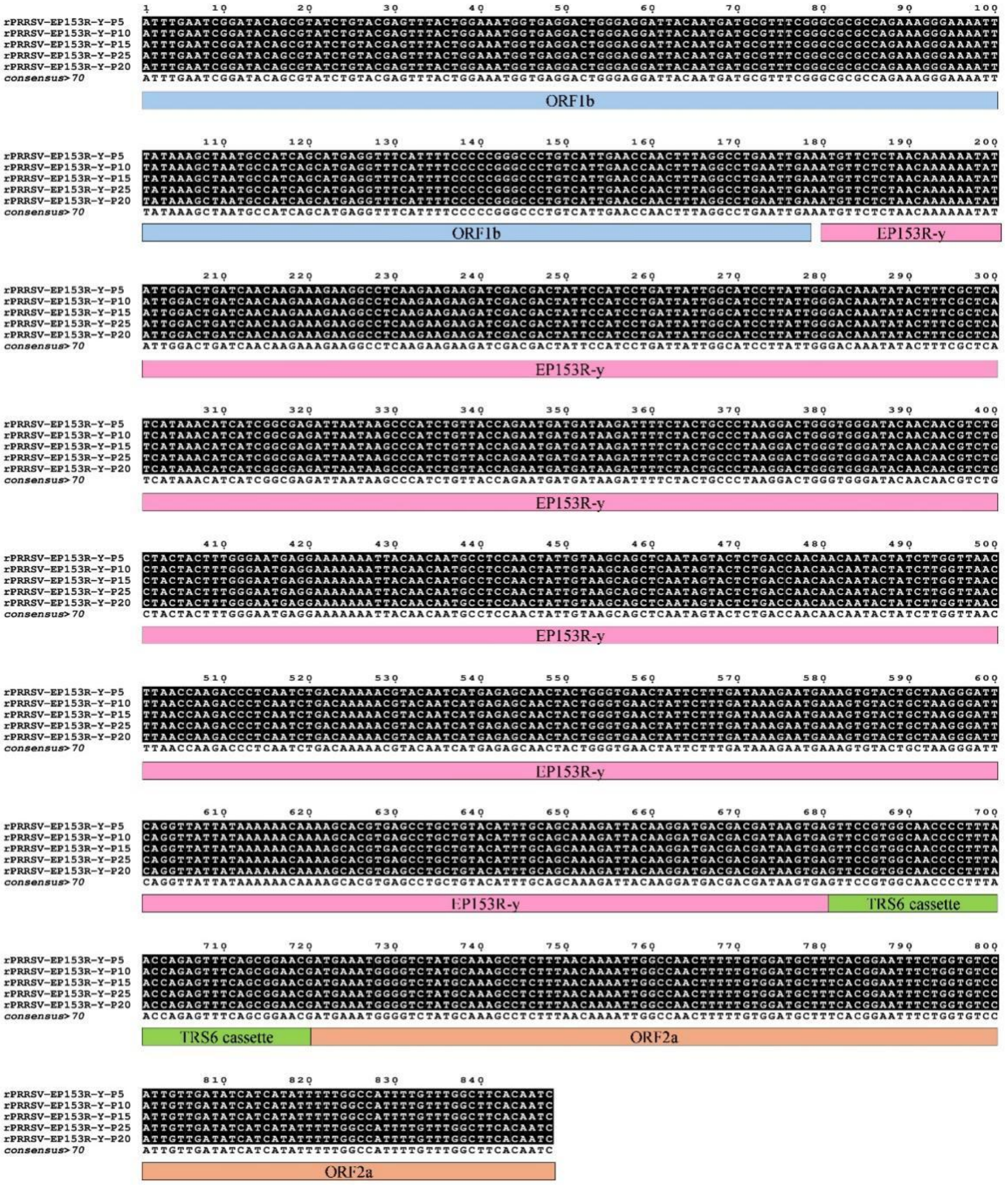

Figure S1. The sequencing results comparison of rPRRSV-EP153R including P5, P10, P10, P20 and P25 generations.

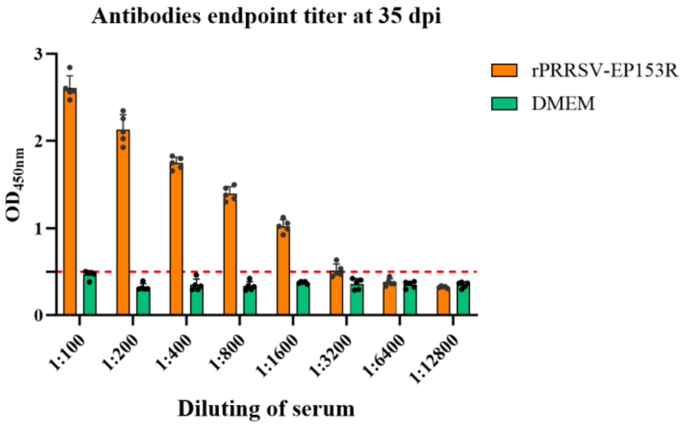

Figure S2. Detection of the endpoint titer of the pig serum antibodies after 35 days post-immunization. Dashed horizontal red line represents the pEP153R-iELISA positive cutoff (OD_{450} = 0.499).
